# Profiles of Emergency Department Users with Psychiatric Disorders Related to Barriers to Outpatient Care

**DOI:** 10.3390/ijerph21020234

**Published:** 2024-02-16

**Authors:** Tiffany Chen, Zhirong Cao, Francine Ferland, Lambert Farand, Marie-Josée Fleury

**Affiliations:** 1 Department of Psychiatry, McGill University, Montreal, QC H3A 1A1, Canada; tiffany.chen2@mail.mcgill.ca; 2 Douglas Hospital Research Centre, Douglas Mental Health University Institute, Montreal, QC H4H 1R3, Canada; zhirong.cao@douglas.mcgill.ca; 3 School of Social Work, Addiction Rehabilitation Centre, Laval University, National Capital University Integrated Health and Social Services Centre, Quebec City, QC G1V 0A6, Canada; francine.ferland.ciussscn@ssss.gouv.qc.ca; 4 Department of Health Administration, Policy, and Evaluation, School of Public Health, University of Montreal, Montreal, QC H3N 1X9, Canada; lambert.farand.umontreal@gmail.com

**Keywords:** mental health, psychiatric disorders, emergency departments, health services, barriers to care, needs assessment

## Abstract

Emergency department (ED) overcrowding is a growing problem worldwide. High ED users have been historically targeted to reduce ED overcrowding and associated high costs. Patients with psychiatric disorders, including substance-related disorders (SRDs), are among the largest contributors to high ED use. Since EDs are meant for urgent cases, they are not an appropriate setting for treating recurrent patients or replacing outpatient care. Identifying ED user profiles in terms of perceived barriers to care, service use, and sociodemographic and clinical characteristics is crucial to reduce ED use and unmet needs. Data were extracted from medical records and a survey was conducted among 299 ED patients from 2021 to 2022 in large Quebec networks. Cluster algorithms and comparison tests identified three profiles. Profile 1 had the most patients without barriers to care, with case managers, and received the best primary care. Profile 2 reported moderate barriers to care and low primary care use, best quality of life, and more serious psychiatric disorders. Profile 3 had the most barriers to care, high ED users, and lower service satisfaction and perceived mental/health conditions. Our findings and recommendations inform decision-makers on evidence-based strategies to address the unmet needs of these vulnerable populations.

## 1. Introduction

Emergency department (ED) overcrowding is a growing problem worldwide [1]. It has been associated with increased waiting times, morbidity and mortality, and decreased quality of care [1,2]. High ED users have historically been targeted to reduce ED overcrowding and associated high costs [3]. Patients with psychiatric disorders, including substance-related disorders (SRDs), are among the largest contributors to high ED use [4], which is usually defined as 4+ ED visits/year—a standard benchmark often used, especially in Canadian studies [4,5]. Across Canada, there is a rising trend in both the overall number of ED visits and visits linked to high ED users [5]. A Quebec study has shown that in 2014–2015, patients with psychiatric disorders used EDs almost twice as often as patients without psychiatric disorders, and 17% of them were high ED users who accounted for close to half of all ED use and hospitalizations [6]. High ED use may be an indicator of unmet needs, and since EDs are not an appropriate setting for treating recurrent patients or for replacing outpatient care, it is important to examine and address the unmet needs of these high ED users. A Canadian study found that approximately 20% of ED visits could be dealt with more efficiently in other settings [7]. However, ED users with psychiatric disorders, including high ED users, are a heterogeneous group that features distinct patient profiles, which suggests that personalized care should be adapted to these patients’ needs. Identifying outpatient service use profiles of patients with psychiatric disorders who use EDs, and integrating barriers to care that explain unmet needs, may thus be key to improving mental health services for these patients and reducing ED use.

Several studies that have assessed determinants of unmet service needs among patients with psychiatric disorders have found that unmet needs were associated with being female [8,9,10], being younger [8], having severe or evolving symptoms [11], or having co-occurring psychiatric disorders or SRDs [10,12,13], and poor physical health [9]. Research in this area has also identified potential barriers to care that explain unmet service needs. Most studies reported motivational or attitudinal barriers [8,9,14,15,16,17,18,19,20,21], while a few reported structural barriers [11,22] as the major reasons underlying perceived unmet needs among adults with psychiatric disorders. Yet, no typology has linked profiles of barriers to care explaining unmet service needs among ED users with psychiatric disorders. The typologies pertaining to ED users with psychiatric disorders have mostly focused on the socio-demographic and clinical characteristics of high ED users, mostly finding them to be young, single, male, and with medical comorbidities, but few typology studies have examined patient service use patterns [23,24]. One recent Canadian study that identified three profiles regarding the quality of outpatient care use for patients with SRDs found that the profile with the most frequent ED use and hospitalizations was made up of high outpatient service users mostly affected by psychiatric disorders and personality disorders [25]. Another study identified three profiles of moderate ED users with psychiatric disorders: one composed of young males with SRDs who were low outpatient service users; one of middle-aged females with common psychiatric disorders; and one of older patients with co-occurring psychiatric disorders–chronic physical illnesses. Patients in these last two profiles mainly consulted general practitioners (GPs). Also identified in that study was a fourth profile of high ED users with multiple psychiatric disorders–SRDs using mostly specialized services [26]. Other studies found that the number of GPs consulted, higher hospitalization and specialized service use were associated with high ED user profiles [24,27].

This study is original in that it considers the number of barriers to care in relation to overall service use patterns among patients with psychiatric disorders, which may explain their psychiatric ED use and inform clinicians and policymakers on how to better respond to the unmet needs of these vulnerable patients and avoid repeated ED use. Most studies have evaluated the presence or absence of unmet needs without examining the number of barriers to care [26,28]. Profiles of ED users in relation to barriers to care, primary care and specialized service use, and patient satisfaction with care have not yet been reported, though they may assist in tailoring more personalized treatment options. Few studies on ED use among patients with psychiatric disorders have integrated medical records from large service networks and patient surveys in order to assess comprehensive data linked to service use and the individual profiles of patients. Through cluster analysis, this study aimed to identify ED user profiles based on the patients’ perceived barriers to outpatient care and service use and to associate these profiles with sociodemographic and clinical characteristics in order to better understand psychiatric ED use and recommend more targeted interventions.

## 2. Materials and Methods

### 2.1. Study Setting and Data Collection

This study was conducted in four ED networks in Quebec, Canada. Patients with a psychiatric disorder, including SRDs, who were 18+ years old were randomly recruited through a list of 1751 ED users identified by the networks’ ED staff. Of the first 563 eligible patients that were reached, 450 (80%) agreed to be referred to the research team to participate in the study. The research coordinator then contacted them to have them complete a 45 min standardized survey by phone, in English or French. The survey questionnaire was validated by a steering committee, including ED clinicians who helped with the research, and it integrated standardized questions from known and published surveys and standardized scales. The surveys were administered by trained interviewers between 1 March 2021 and 13 May 2022, and they were closely monitored by the research team. Participants also had to allow the research team to access their medical records, which were merged with the survey results. The survey and medical records collected data for the 12 months preceding patient interviews, except for recurrent ED use, which was measured over the 2 years prior to this 12-month period. Medical records reported patient data related to ED use (BDCU databases), hospitalization or inpatient care (MED-ECHO databases), specialized psychiatric disorder care provided by biopsychosocial teams (outpatient hospital databases), and psychosocial primary care mental health services provided in community healthcare centers (I-CLSC databases). The BDCU and MED-ECHO databases provided patient health diagnoses based on the International Classification of Diseases, Tenth Revision (ICD-10) (Table S1). Each database included information on patient service use (e.g., type, frequency), but only within the ED networks and in public organizations (hospitals, community healthcare centers). The survey questionnaire completed the information extracted from the databases, namely service use outside the ED networks and in non-public services (e.g., community-based services: crisis centers). Patient profiles considered barriers to outpatient care related to unmet needs and other service use variables. Patient sociodemographic and clinical characteristics were then associated with these patient profiles. Participation in the study was voluntary. Patients who consented received a compensation of CAD $20 for participating. Ethics approval was granted by the Douglas Mental Health University Institute ethics committee (IUSMD 20-26).

### 2.2. Study Variables

Variables considered for creating patient profiles specifically included the following: number of barriers to outpatient care, having a case manager, number of consultations with GP (0, 1–4, 5+), number of primary care service uses other than with GP (0, 1–4, 5+), number of specialized outpatient care uses (0, 1–4, 5+), satisfaction with outpatient services, high ED use, and high hospitalization, measured for the 12 previous months. Also included was the number of recurrent ED users, which was measured for the preceding 13–36 months. Barriers to outpatient care refers to health system features and individual characteristics or behaviors related to the patients’ unmet needs, unmet needs being defined as “the difference between services judged necessary to appropriately deal with health problems, and services actually received” [29]. Based on a question used in the Canadian Community Health Survey (CCHS) [30], patients were asked on a 5-point scale if services provided outside of EDs responded to their needs. If they answered between 1 (totally disagree) and 3 (somewhat agree), they were then asked to identify barriers to outpatient care, with 13 possible choices that could be associated with motivational barriers (e.g., “I prefer to manage by myself”; “have not gotten around to it (e.g., too busy)”;) or structural barriers (e.g., “Help is not readily available”; “do not know how or where to get this kind of help”). The number of barriers to outpatient care was logged for each patient (0, 1–2, 3+). The variable “having a case manager” was also measured as it plays a key role in responding to patients with complex needs and helping them navigate the health and social services system [31]. Having a case manager has been proven to help patients reduce acute care [32,33,34]. The number of consultations with GPs included care provided by family doctors and GPs working in walk-in clinics. The number of primary care service uses other than with GPs referred to services provided by psychologists in private practice, community healthcare centers mainly dispensing psychosocial services, and community-based organizations (e.g., suicide prevention centers). The number of specialized outpatient care services included hospital psychiatric services integrating treatment from psychiatrists and their teams, assertive community treatment and intensive case management programs, and services from addiction treatment centers. Satisfaction with outpatient services represented the mean score of patient satisfaction with each of the outpatient services they used, evaluated on a 5-point scale, with higher scores indicating greater satisfaction. High ED use was defined as using EDs 4+ times/year. Patients in this study were categorized as low ED users (1–3 visits/year) or high ED users (4+ visits/year) [35,36]. Recurrent high ED users were categorized as 8+ visits over the preceding 13–36 months. High hospitalization was defined as being hospitalized 3+ times/year [37].

Sociodemographic characteristics were measured for the 12 months preceding interviews and included sex, age group (16–20, 30–49, 50+), civil status (single, in a relationship), stigma, and quality of life. Stigma was measured on a 5-point scale, with higher scores indicating less stigma, through the following CCHS question: “Most people in my community treat a person with a psychiatric disorder, including a SRD, in the same manner as they would treat any other person [30].” Quality of life was assessed on a 7-point scale using the Satisfaction with Life Domains Scale, which comprises 20 items organized in 5 domains (e.g., daily living and social relationships), with higher scores indicating higher quality of life [38].

Clinical characteristics were also measured for the preceding 12 months and included psychiatric disorders, suicidal behaviors (suicide attempt or ideation), perceived mental/physical health conditions, co-occurring psychiatric disorders–SRDs or psychiatric disorders–chronic physical illnesses, and percentage of high priority in ED triage. Psychiatric disorders included serious psychiatric disorders (schizophrenia spectrum and other psychotic disorders; bipolar disorders), personality disorders, and common psychiatric disorders (anxiety, depressive and adjustment disorders; attention-deficit/hyperactivity disorder). SRD included alcohol- and drug-related disorders (use, induced, intoxication, and withdrawal). In addition to medical records, the Alcohol Use Disorders Identification Test [39] and the Drug Abuse Screening Test-20 [40] were used to identify SRDs, as these disorders are often underdiagnosed in medical records [41]. Based on the merging of two CCHS questions (“How do you see your ‘physical’ and ‘mental health’ conditions”), perceived physical/mental health conditions were measured on a 10-point scale, with 7+ indicating better-perceived health conditions. Chronic physical illnesses were identified based on an adapted version integrating both the Charlson and Elixhauser Comorbidity indexes [42]. ED triage priority was based on the Canadian Triage Acuity Scale [43] which consists of 5 priority levels or illness severities, with levels 4–5 considered treatable in outpatient care. In this study, ED use with high triage priority (1–3) was considered a proxy for functional disability, based on the mean number of ED visits per patient with 1–3 triage priority divided by the total of ED visits per patient (1–5). 

### 2.3. Analysis

Univariate analyses consisted of frequency distributions for categorical variables and mean values with standard deviations for continuous variables. Missing values (less than 1%) were randomly distributed and imputed by mean and mode. The k-means cluster algorithm with the Gower dissimilarity coefficient [44] was used to identify ED user profiles. Several k-means solutions with different numbers of profiles were computed for the cluster analysis to determine the optimal number of patient profiles. The three-profile solution had the largest Calinski–Harabasz pseudo-F value [45], indicating it was the most distinct result. To determine statistical differences between the profiles, pairwise comparisons were conducted using chi-square tests or Fisher’s exact tests for categorical variables, and *t*-tests or Wilcoxon rank-sum tests for continuous variables. Analyses were performed with Stata 17.

## 3. Results

Of the 450 ED users referred by ED staff, 50 could not be reached and 300 participated, for a response rate of 75%. One patient withdrew, resulting in a final sample size of 299 patients. Most participants (55%) were women, 69% aged 30+ years, 82% single, 50% perceived high stigma, and the mean score for patient quality of life was 4.55 out of 7 (Table 1). Over half the participants (59%) had SRDs, 57% had common psychiatric disorders, 44% had serious psychiatric disorders, 42% had personality disorders, while 54% had suicidal behaviors, and 38% and 40% had co-occurring psychiatric disorders–SRDs or psychiatric disorders–chronic physical illnesses, respectively. Only 32% perceived having good physical/mental health conditions (score of 7+ out of 10), and 59% reported a high percentage of high triage priority (67–100%) for their ED use. Barriers to outpatient care were identified by 37% of patients, with 15% reporting 3+ barriers. Meanwhile, 58% had a case manager, 71% had consulted a GP, 58% had used 5+ primary care services other than GPs, and 38% reported using 5+ specialized outpatient services. While the mean score for satisfaction with outpatient services was 4.02 out of 5, 61% of the patients were high ED users, 40% were high recurrent ED users, and 21% had been hospitalized at least three times in the 12 months prior to their interview.

### 3.1. Patient Profiles Related to Barriers to Outpatient Care and Service Use

Three patient profiles were identified (Table 2). Accounting for 50% of the sample, Profile 1 included the most patients without barriers to outpatient care (87%) compared to Profiles 2 (68%) and 3 (0%). This profile consisted of the most patients who had a case manager (71%) compared to Profiles 2 (41%) and 3 (51%), and it had the most patients (85%) with 5+ primary care service uses other than GPs per year, comparable to Profile 3 (75%) but much higher than Profile 2 (0%). Profile 1 also included fewer high ED users (58%) and recurrent high ED users (37%) than Profile 3 (87% and 57%, respectively). Profile 1 also reported the highest satisfaction with outpatient services (4.23/5), similar to Profile 2 (4.07/5) but significantly higher than Profile 3 (3.46/5). Profile 1 was labeled as follows: Patients with low barriers to outpatient care and high primary care service use, with most having a case manager.

Accounting for 29% of the sample, Profile 2 had the fewest patients (41%) that were being followed by a case manager, a result comparable to Profile 3 (51%). More Profile 2 patients had not consulted a GP (56%) or used other primary care services (83%) than those in Profiles 1 (17%, 0%) and 3 (19%, 3%). Profile 2 had a lower number of high ED users (47%) than Profile 3 (87%) but was fairly comparable in that respect to Profile 1. Profile 2 also had a lower number of recurrent high ED users (30%) than Profile 3. Profile 2 was labeled as follows: Patients with moderate barriers to outpatient care and low primary care service use.

Accounting for 21% of the sample, all Profile 3 patients (100%) reported barriers to outpatient care, with a higher percentage of them (56%) experiencing 1–2 or 3+ barriers than in other profiles (13% and 0%, respectively, in Profile 1; 32% and 13% in Profile 2). With 44% of patients reporting 5+ consultations a year with GPs, Profile 3 was the highest in that regard, followed relatively closely by Profile 1. Profile 3 patients reported the lowest satisfaction with outpatient services (3.46/5) compared to Profiles 1 (4.23/5) and 2 (4.07/5). Profile 3 also had the highest number of high ED users (87%). Compared to Profiles 1 (37%) and 2 (30%), Profile 3 also had the greatest number of recurrent high ED users (57%). Profile 3 was labeled as follows: Patients with high barriers to outpatient care and high service use, including high and recurrent high ED use, and not satisfied with service use.

### 3.2. Associations between Patient Profiles and Covariates

Fewer patients in Profile 1 perceived high mental health stigma (46%) than those in Profile 3, and they had less serious psychiatric disorders (39%) but more common psychiatric disorders (60%) and suicidal behaviors (57%) than Profile 2 (Table 3). More Profile 1 patients (32%) perceived good physical/mental health conditions than in Profile 3. Their quality-of-life score (4.54/7) was higher than that of Profile 3 but lower than Profile 2. Profile 1 also had fewer patients with low ED triage priority (12%) compared to Profile 2 (26%). Profile 2 included fewer women (45%) and fewer patients with personality disorders (32%) and co-occurring psychiatric disorders–chronic physical illnesses (29%), and more of them perceived good physical/mental health conditions (43%) compared to Profile 3. Profile 2 also reported fewer common psychiatric disorders (45%) and suicidal behaviors (39%), and more ED users with lower triage priority (26%) and a better quality of life (4.83/7) than the other two profiles; however, Profile 2 had more patients with serious psychiatric disorders (56%) than Profile 1. Profile 3 included more women (63%), personality disorders (63%), common psychiatric disorders (63%), suicidal behaviors (67%), and co-occurring psychiatric disorders–SRDs and physical illnesses (54%), but fewer ED users with lower triage priority (11%) than Profile 2. More Profile 3 patients perceived high stigma (65%) compared to Profile 1, and fewer of them perceived good physical/mental conditions (16%) and high quality of life (4.83/7) than in other profiles.

## 4. Discussion

Three profiles of patients with different barriers to care and service use among ED users were identified. Out of 299 patients, barriers to outpatient care explaining unmet needs were identified by 37% of patients who use EDs. This percentage is similar to that found in studies on unmet needs among patients with psychiatric disorders (27%) [46] but lower than among patients with SRDs (82%) [47] and the homeless (89%) [48]—though it is higher than the percentage in the general population (22%) [49]. The fact that 61% of study patients were high ED users and 40% were recurrent high ED users might explain their high number of perceived barriers to care. Loneliness, elevated perceived stigmatization and health issues might also explain unmet needs, even if the majority of our study patients had made substantial use of outpatient services and had a case manager.

It is interesting to note that 87% of Profile 1 patients, who accounted for half of our sample, reported no barriers to outpatient care. This could easily be explained by their high use of primary care services and the fact that over two-thirds of them had a case manager. Profile 1 had the most patients with 5+ primary care service uses per year other than GPs. Having a regular source of care and receiving biopsychosocial services were both previously associated with fewer unmet needs [50,51]. Case management is known to be successful in helping patients access outpatient services that adequately respond to their needs [52]. Comparable to Profile 3, Profile 1 patients mostly had common psychiatric disorders, which explains their high primary care use. Primary care settings often serve as the first and only point of contact for individuals experiencing common psychiatric disorders [53]. More patients in Profile 1 perceived having good physical/mental health conditions and quality of life than those in Profile 3, with fewer reporting high stigma. This may explain the low number of barriers to care reported by Profile 1 patients. According to the literature, fewer unmet needs or barriers to care were associated with higher self-rated health and quality of life [52]. To reduce the ED use of Profile 1 patients, better access to primary care and care coordination could be improved. Previous studies have shown that continuity of primary care, such as better access to after-hours primary care, may reduce non-urgent ED utilization [53,54]. Collaborative care management has also been shown to improve outcomes for patients with common psychiatric disorders and help lower ED visits and other acute care use [55,56].

Accounting for one-fifth of the sample, Profile 3 had the most barriers to outpatient care despite their high service use. Compared to Profile 1, fewer of them reported having a case manager or using primary care services other than GPs; they also showed the highest number of high ED and recurrent high ED users and reported the least satisfaction with services compared to Profiles 1 and 2. These service patterns may easily explain the higher number of barriers to outpatient care seen in Profile 3. High and recurrent high ED use were previously found to be linked to insufficient or inadequate outpatient care [57]. The fact that patients who were less satisfied with service use reported more barriers to care was not surprising, as satisfaction with care is a key patient outcome [58]. Profiles with more service use (1 and 3) also included more women, who are known to use mental health services more readily than men [59,60]. Though Profiles 3 and 1 shared very similar sociodemographic and clinical characteristics, Profile 3 patients perceived their mental/physical health conditions and quality of life as the worst of the three profiles, and they felt more stigmatized than those in Profile 1. Profile 3 also had more patients with personality disorders and suicidal behaviors than Profile 2. The associations between poorer patient perception of their conditions and greater unmet needs [61], and the fact that patients with personality disorders have higher ED use and are less satisfied with outpatient services, are all well documented [62]. As the ED is often used for addressing crisis situations [63], finding that these patients had more barriers to care due to greater suicidal behaviors and higher triage priority was not astonishing, especially since it has previously been reported in the literature [64,65]. To reduce high ED use among Profile 3 patients, assertive community treatment (ACT) might be delivered to them or dialectical behavioral therapy (DBT) made available to those with personality disorders. ACT is a program offered to adults with severe psychiatric disorders requiring very intensive services. Each patient is treated by an interdisciplinary healthcare team that offers specialized services at the treatment intensity each patient needs [66]. The literature has strong overall evidence for reducing acute care use in patients who received ACT when compared to usual care [67]. DBT, an evidence-based psychotherapy approach, has also been recommended as a first-line treatment for the prevention of suicidal behaviors and psychiatric ED use in diverse clinical populations, including those with personality disorders and high-risk and acutely suicidal clients [68,69].

One-third of Profile 2 patients experienced moderate barriers to outpatient care. Of all the profiles, Profile 2 showed the lowest use of primary care services, but its high ED use and elevated satisfaction with care were similar to Profile 1. Over half of the Profile 2 patients were men; they had the highest percentage of serious psychiatric disorders but the lowest ED triage priority, the least suicidal behaviors, and the highest quality of life. Previous literature has shown that men are less likely than women to seek mental health treatments, or that they will seek help only as a last resort [70]. This lack of help-seeking behavior has been associated with traditional masculine norms such as being strong and self-reliant, and men may therefore inadvertently downplay potentially serious medical or mental conditions. This is underscored by the fact that the men in Profile 2 were the majority and had the highest percentage of serious psychiatric disorders but were still triaged at lower priority [71]. Their high ED use with lower triage priority could also be explained by the fact that patients with serious psychiatric disorders are reported to receive less primary care than patients with common psychiatric disorders [72]. Primary care providers, most notably GPs, are said to be less comfortable in treating patients with serious psychiatric disorders [73]. In line with the recovery movement, patients with serious psychiatric disorders can lead a good life despite their chronic illness as they often carry fewer expectations, especially if they receive the help they need [74]. Indeed, studies have shown that patients with serious psychiatric disorders usually demonstrate better quality of life compared to those with common psychiatric disorders [74]. Of all the profiles, Profile 2 patients reported the best quality of life and perceived mental/physical conditions, along with the least high ED triage priority, all of which may explain why they perceived moderate barriers to care. ED use may be reduced for Profile 2 patients by providing them with more intensive case management (ICM), including better access to primary care. ICM is a community-based package of care aiming to provide long-term care for people with serious psychiatric disorders who do not require immediate admission. Intensive case management has been previously documented as an effective means of reducing ED use by high ED users [75]. Additionally, a few studies have evaluated the effects of enhanced primary care on the ED use of patients with serious psychiatric disorders and found that enhanced primary care reduced ED utilization over time [76]. 

This study has a few limitations. First, even though we used the unmet needs and barriers-to-care questions found in the CCHS, unmet needs were not measured with a standardized scale and were self-reported. Second, the number of barriers to care was examined, but we did not investigate types of care (e.g., information, counseling, medication) or types of barriers to care (motivational vs. structural). Moreover, as in all survey studies, it is subject to participation bias and the subjectivity associated with “perceived” barriers to care. Lastly, patients were recruited from large urban psychiatric ED networks in a public healthcare system, so study findings may not be generalizable to other types of EDs, territories, or contexts.

## 5. Conclusions

To our knowledge, this study was the first to identify profiles of ED users in terms of their perceived barriers to outpatient care, service use, and associated sociodemographic and clinical characteristics. Three profiles were found. Comprising half of the sample, Profile 1 had the most patients without barriers to outpatient care or unmet needs, receiving the best primary care services, with a majority of them having a case manager. Representing a third of the sample, Profile 2 reported moderate barriers to outpatient care and low primary care service use; it included more patients with serious psychiatric disorders and who reported the best quality of life. Accounting for one-fifth of the sample, Profile 3 had the most barriers to outpatient care and the greatest percentages of high and recurrent high ED users; these patients were the least satisfied with services and reported the worst perceived mental/health conditions and quality of life. For Profiles 1 and 2, collaborative and integrative care models may better support primary care providers in treating people with common and serious psychiatric disorders. Moreover, the greater availability of family physicians and case managers may improve the overall responsiveness of primary and ambulatory services to offer better care alternatives than ED use for urgent mental health conditions. Strategies such as ACT, integrated co-occurring treatment, and shared care between psychiatrists and primary care services may also be implemented to improve the adequacy of care for patients like those in Profile 3, who have complex clinical conditions.

## Figures and Tables

**Table 1:** Characteristics of patients (*N* = 299).

	N/mean	%/SD.
Sociodemographic characteristics		
(measured over the preceding 12 months) ^a^		
Sex		
Women	165	55.18
Men	134	44.82
Age		
16–29 years	92	30.77
30–49 years	117	39.13
50+ years	90	30.1
Civil status		
Single (including separated, divorced, or widowed)	246	82.27
In a relationship	53	17.73
Stigma		
High	149	49.83
Median	54	18.06
Low	96	32.11
Quality of life (mean/SD)	4.55	1.06
Clinical characteristics (measured over the preceding 12 months)		
Serious psychiatric disorders	133	44.48
Personality disorders	127	42.47
Common psychiatric disorders	169	56.52
Substance-related disorders (SRDs)	175	58.53
Suicidal behaviors (suicide attempt or ideation)	161	53.85
Good perceived mental/physical health conditions (7+)	95	31.77
Co-occurring psychiatric disorders–SRD	113	37.79
Co-occurring psychiatric disorders–chronic physical illnesses ^b^	119	39.8
Percentage of high priority in emergency department (ED) triage (1, 2 and 3)		
0–33%	48	16.06
34–66%	77	25.75
67–100%	174	58.19
Service use patterns (measured over the preceding 12 months, or other as specified)		
Number of barriers to outpatient care		
0	188	62.88
1–2	65	21.74
3+	46	15.38
Having a case manager	174	58.19
Number of consultations with general practitioners (GPs)		
0	87	29.1
1–4	124	41.47
5+	88	29.43
Number of primary care service uses other than GPs		
0	74	24.75
1–4	51	17.06
5+	174	58.19
Number of specialized outpatient care use		
0	87	29.1
1–4	98	32.78
5+	114	38.12
Satisfaction with outpatient services (mean/SD)	4.02	0.76
High ED use (4+)	182	60.87
Recurrent high ED users (8+) (measured over the preceding 13–36 months)	117	39.13
High hospitalization (3+)	63	21.07

^a^ All variables are defined in the Methods section of the manuscript. For the list of diagnostics, refer to Table S1. ^b^ Chronic physical illnesses included chronic pulmonary disease, cardiac arrhythmia, tumor with or without metastasis, renal disease, fluid electrolyte disorder, myocardial infarction, congestive heart failure, metastatic cancer, dementia, stroke, neurological disorder, liver disease, pulmonary circulation disorder, coagulopathy, weight loss, paralysis, AIDS/HIV.

**Table 2:** Patient profiles using emergency department (ED) based on barriers to care and service use (N = 299).

	Profile 1 *	Profile 2 *	Profile 3 *
Group Size: N (%)	148 (49.83%)	87 (29.10%)	63 (21.07%)
	%/mean	%/mean	%/mean
Service use (measured over the preceding 12 months, or other as specified) ^a^			
Number of barriers to outpatient care			
0	86.58 ^2,3^	67.82 ^1,3^	0.00 ^1,2^
1–2	13.42	19.54	44.44
3+	0.00	12.64	55.56
Having a case manager	71.14 ^2,3^	41.38 ^1^	50.79 ^1^
Number of consultations with general practitioners (GPs)			
0	17.45 ^2^	56.32 ^1,3^	19.05 ^2^
1–4	45.64	37.93	36.51
5+	36.91	5.75	44.44
Number of primary care service uses other than with GPs			
0	0.00 ^2,3^	82.76 ^1,3^	3.17 ^1,2^
1–4	14.77	17.24	22.22
5+	85.23	0.00	74.61
Number of specialized outpatient care use			
0	25.51	34.48	30.16
1–4	32.21	35.63	30.16
5+	42.28	29.89	39.68
Satisfaction with outpatient services (mean/SD/ maximum 5)	4.23 (0.62) ^3^	4.07 (0.80) ^3^	3.46 (0.74) ^1,2^
High ED use (4+/year)	57.72 ^3^	47.13 ^3^	87.30 ^1,2^
Recurrent high ED users (8+) (measured over the preceding 13–36 months)	36.91 ^3^	29.89 ^3^	57.14 ^1,2^
High hospitalization (3+)	18.12	21.84	26.98

^a^ All variables are defined in the Methods section of the manuscript. ^1,2,3^ Superscript numbers indicate significant differences between profiles at *p* < 0.05. * Profile 1: Patients with low barriers to outpatient care and high primary care service use, with most having a case manager. * Profile 2: Patients with moderate barriers to outpatient care and low primary care service use. * Profile 3: Patients with high barriers to outpatient care and high service use, including high and recurrent high ED use, and not satisfied with service use.

**Table 3:** Associations between patient profiles and covariates (N = 299).

	Profile 1 *	Profile 2 *	Profile 3 *
Group size: N (%)	148 (49.83%)	87 (29.10%)	63 (21.07%)
	%/mean	%/mean	%/mean
Sociodemographic characteristics (measured over the preceding 12 months) ^a^			
Sex			
Women	57.72	44.83 ^3^	63.49 ^2^
Men	42.28	55.17	36.51
Age			
16–29 years	26.85	33.33	36.51
30–49 years	38.26	40.23	39.68
50+ years	34.89	26.44	23.81
Civil status			
Single (including separated, divorced, or widowed)	81.21	79.31	88.89
In a relationship	18.79	20.69	11.11
Stigma			
High (1–2 scores)	45.64 ^3^	45.98	65.08 ^1^
Median (3 score)	19.46	18.39	14.29
Low (4–5 scores)	34.90	35.63	20.63
Clinical characteristics (measured over the preceding 12 months)			
Serious psychiatric disorders	38.93 ^2^	56.32 ^1^	41.27
Personality disorders	39.60	32.18 ^3^	63.49 ^2^
Common psychiatric disorders	60.40 ^2^	44.83 ^1,3^	63.49 ^2^
Substance-related disorders (SRDs)	53.69	65.52	60.32
Suicidal behaviors (suicide attempt or ideation)	57.05 ^2^	39.08 ^1,3^	66.67 ^2^
Good perceived mental/physical health conditions (7+/ maximum 10)	32.21 ^3^	42.53 ^3^	15.87 ^1,2^
Co-occurring psychiatric disorders–SRDs	36.24	39.08	39.68
Co-occurring psychiatric disorders–chronic physical illnesses ^b^	40.27	28.74 ^3^	53.97 ^2^
Percentage of high priority in emergency department (ED) triage (1, 2 and 3/out of 5)			
0–33%	12.08 ^2^	26.44 ^1,3^	11.11 ^2^
34–66%	27.52	19.54	30.16
67–100%	60.40	54.02	58.73
Quality of life (mean/SD, maximum 7)	4.54 (0.94) ^2,3^	4.83 (1.14) ^1,3^	4.19 (1.11) ^1,2^

^1,2,3^ Superscript numbers indicate significant differences between profiles at *p* < 0.05. ^a^ All variables are defined in Section 2 of the manuscript. For the list of diagnostics, refer to Table S1. ^b^ See footnote ^b^ in Table 1. * See * Profiles footnotes in Table 2.

## Data Availability

In accordance with the applicable ethics regulations in the province of Quebec, the authors do not have permission to share the data extracted from databases used for this study.

## Supplementary Materials

The following supporting information can be downloaded at https://www.mdpi.com/article/10.3390/ijerph21020234/s1, Table S1: Codes for psychiatric disorders including substance-related disorders and chronic physical illnesses according to the International Classification of Diseases, Tenth revision.
