# Supplementary material for: Profiles of Emergency Department Users with Psychiatric Disorders Related to Barriers to Outpatient Care
Source: Int J Environ Res Public Health. 2024 Feb 16;21(2):234. doi: 10.3390/ijerph21020234 (PMC10888102; doi:10.3390/ijerph21020234)
Supplement: Supplementary file 1 [file ijerph-21-00234-s001.zip › ijerph-2846589-supplementary.pdf]

Table S1

Codes for psychiatric disorders including substance-related disorders and chronic physical illnesses according to the International Classification of Diseases, Tenth revision

**Diagnoses**	***International Classification of Diseases, Tenth Revision, Canada*** **(*ICD-10-CA)***
**Psychiatric disorders** [a]	
Serious Psychiatric disorders	
Schizophrenia spectrum and other psychotic disorders	F20* (schizophrenic disorders); F22* (persistent delusional disorders); F23 (acute and transient psychotic disorders); F24* (induced delusional disorder); F25* (schizoaffective disorders); F28* (other psychotic disorder not due to a substance or known physiological condition); F29* (unspecified psychosis not due to a substance or known physiological condition); F448 (other dissociative and conversion disorders); F481 (depersonalization - derealization syndrome)
Bipolar disorders	F300-F302, F308, F309 (manic episode); F310-F317, F318, 319 (bipolar episode)
Personality disorders	F600 (paranoid personality disorder); F61 (mixed and other personality disorders); F340 (cyclothymic disorder); F341 (dysthymic disorder); F601 (schizoid personality); F603 (borderline personality disorder); F605 (obsessive-compulsive personality disorder); F604 (histrionic personality disorder); F607 (dependent personality disorder); F602 (antisocial personality disorder); F609 (unspecified personality disorder); F21 (schizotypal personality); F606 (avoidant personality disorder); F608 (other specified personality disorders); F681 (factitious disorder); F688 (other specified disorders of adult personality and behaviour); F69 (unspecified disorder of adult personality and behaviour)
Common Psychiatric disorders	
Depressive disorders	F320- F323 (major depressive disorder, single episode); F328 (other depressive episodes); F329 (depressive episode, unspecified); F330-F334 (major depressive disorder, recurrent); F338 (other recurrent depressive disorders); F339 (recurrent depressive disorder, unspecified); F348 (other persistent mood [affective] disorders); F380, F381 (persistent mood [affective] disorder, unspecified); F388 (other specified mood [affective] disorders); F39 (unspecified mood [affective] disorders); F412* (mixed anxiety and depressive disorder)*
Anxiety disorders	F40 (phobic anxiety disorders); F41(other anxiety disorders); F42 (obsessive-compulsive disorder); F45 (somatoform disorders); F48 (other neurotic disorders); F93, F94 (disturbance of emotions specific to childhood and adolescence)
Adjustment disorders	F430 (acute stress reaction); F431 (post-traumatic stress disorder); F432 (adjustment disorders); F438 (other reactions to severe stress); F439 (reaction to severe stress, unspecified)
Attention deficit/hyperactivity disorder	F900; F901; F908; F909 (attention deficit/hyperactivity disorder)
**Suicide attempt** [a, b]	X60-Y09, Y870, Y871, Y35-Y36, Y890, Y891
**Substance-related disorders** [a]	

Alcohol-related disorders	F101*, F102* (alcohol abuse or dependence); F103, F104* (alcohol withdrawal); F105-F109, K700*-K704*, K709*, G621*, I426, K292*, K852, K860, E244, G312, G721, O354 (alcohol-induced disorders); F100*, T510, T511*, T518, T519 (alcohol intoxication)
Cannabis-related disorder	F121, F122 (cannabis abuse or dependence); F123-F129 (cannabis-induced disorders); F120, T407 (cannabis intoxication)
Drug-related disorders other than cannabis	F111, F131, F141, F151, F161, F181, F191, F112, F132, F142, F152, F162, F182, F192 (drug abuse or dependence); F113-F114, F133-F134, F143-F144, F153-F154, F163-F164, F183-F184, F193-F194 (drug withdrawal) F115-F119, F135-F139, F145-F149, F155-F159, F165-F169, F185-F189, F195-F199 (drug-induced disorders); F110, F130, F140, F150, F160, F180, F190, T400-T406, T408, T409, T423, T424, T426, T427, T435, T436, T438, T439, T509, T528, T529 (drug intoxication)
**Chronic physical illnesses** [a, c]	
Renal failure	I120, I131, N18, N19, N250, Z49, Z940, Z992
Cerebrovascular illnesses	G45, G46, I60-I69
Neurological illnesses	G10–G12, G13, G20, G21–G22, G254, G255, G312, G318, G319, G32, G35, G36, G37, G40, G41, G931, G934, R470, R56
Endocrine illnesses (hypothyroidism; fluid electrolyte disorders and obesity)	E00, E01, E02, E03, E890; E222, E86, E87; E66
Any tumor with or without metastasis (solid tumor without metastasis; lymphoma)	C00–C26, C30–C34, C37–C41, C43, C45-C58, C60–C76, C77-C79, C80; C81-C85, C88, C900, C902, C96
Chronic pulmonary illnesses	I278, I279, J40-J47, J60-J64, J65, J66, J67, J684, J701, J703
Diabetes complicated and uncomplicated	E102-E108, E112-E118, E132-E138, E142-E148; E100, E101, E109, E110, E111, E119, E130, E131, E139, E140, E141, E149
Cardiovascular illnesses (congestive heart failure; cardiac arrhythmias; valvular illnesses; peripheral vascular illnesses; myocardial infarction; hypertension and pulmonary circulation illnesses)	I099, I110, I130, I132, I255, I420, I425–I429, I43, I50, P290; I441–I443, I456, I459, I47–I49, R000, R001, R008, T821, Z450, Z950; A520, I70-I72, I730, I731, I738, I739, I771, I790, K551, K558, K559, Z958, Z959; I05–I08, I091, I098, I34–I39, Q230–Q233, Q238, Q239, Z952, Z953, Z954I210-I214, I219, I220, I221, I228, I229, I252; I101, I100, I11, I1500, I1501, I1510, I1511, I1521, I1581, I1590, I1591, I674; I26, I27, I280, I288, I289
Other chronic physical illness categories (blood loss anemia; ulcer illnesses; liver illnesses; AIDS/HIV; rheumatoid arthritis/collagen vascular illnesses, coagulopathy; weight loss, paralysis; deficiency anemia)	D500; K257, K259, K267, K269, K277, K279, K287, K289; B20-B24; D65–D68, D691, D693-D696; B18, I85, I864, I982, K700- K703, K709 K711, K713–K715, K716, K717, K721, K729, K73, K74, K754, K760, K761, K763, K764, K765, K766, K768, K769, Z944; L900, L940, L941, L943, M05, M06, M08, M120, M123, M30, M31, M32–M35, M45, M460, M461, M468, M469; G041, G114, G80, G81, G82, G83; E40–E46, R634, R64, D51–D53, D63, D649; D501, D508; D509

[a] The Canadian Tenth Revision (ICD-10-CA) was used in MED-ECHO (*Maintenance et exploitation des données pour l'étude de la clientèle hospitalière*, hospitalization database) and in BDCU (*Banque de données communes des urgences*, emergency department (ED) database). Diagnoses related to the two databases were considered,

and all data were integrated each year, for each patient. MED-ECHO includes several diagnoses: primary diagnosis and numerous secondary diagnoses. For the databases used in this study, psychiatric disorders were considered as primary diagnoses only, but substance-related disorders (SRD) as both primary and secondary diagnoses, considering that SRD are often underdiagnosed. [b] Diagnostic codes for suicide attempt were registered in the MED-ECHO database. ED use for reasons of suicide ideation or attempt were reported by triage nurses in ED and registered in the BDCU database (and in the survey questionnaire). As they are not diagnostic codes, they were not reported in this table. [c] The list of chronic physical illnesses is based on an adapted and validated version of the Elixhauser Comorbidity Index, integrating the Charlson Index, which consists of 32 major categories of physical illnesses (see reference in the Methods section). For this list of chronic physical illnesses, three categories of psychiatric disorders and two categories of SRD (identified with an asterisk [*]) were also included under psychiatric disorders-SRD, thus appearing twice.
